# Multisystem Inflammatory Syndrome in Children: Unique Disease or Part of the Kawasaki Disease Spectrum?

**DOI:** 10.3389/fped.2021.680813

**Published:** 2021-06-04

**Authors:** Caterina Matucci-Cerinic, Roberta Caorsi, Alessandro Consolaro, Silvia Rosina, Adele Civino, Angelo Ravelli

**Affiliations:** ^1^Dipartimento di Neuroscienze, Riabilitazione, Oftalmologia, Genetica e Scienze Materno-Infantili, Università degli Studi di Genova, Genoa, Italy; ^2^Unità Operativa Complessa (UOC) Clinica Pediatrica e Reumatologia, Istituto di Ricovero e Cura a Carattere Scientifico (IRCCS) Istituto Giannina Gaslini, Genoa, Italy; ^3^Unità di Reumatologia e Immunologia Pediatrica, Ospedale Vito Fazzi, Lecce, Italy; ^4^Sechenov First Moscow State Medical University, Moscow, Russia

**Keywords:** Kawasaki disease, multisystem inflammatory syndrome in children, COVID-19, MIS-C, SARS-CoV-2, toxic shock syndrome, macrophage activation syndrome

## Abstract

One of the most intriguing and mysterious phenomena observed during the COVID-19 pandemic has been represented by the occurrence of the multisystem inflammatory syndrome in children and adolescents (MIS-C). Patients with this condition have some overlapping signs and symptoms with those of Kawasaki disease (KD), but also display clinical features that are uncommon or less frequent in this illness, such as diarrhea, abdominal pain and myocardial involvement. The sickest patients may develop multiorgan failure and shock, usually due to myocarditis. Management is based on the administration of intravenous immunoglobulin, glucocorticoids and, in the most severe instances, anakinra. It is still debated whether MIS-C and KD represent different illnesses or are part of the same disease spectrum. The aim of the present review is to analyze critically the evidence in favor of the latter hypothesis and to provide the authors' personal interpretation of the relationship between the two conditions.

## Introduction

One of the most challenging and enigmatic phenomena observed during the COVID 19 pandemic has been the emergence of the multisystem inflammatory syndrome in children (MIS-C) (1–7). The presenting signs and symptoms were a mix of those of Kawasaki disease (KD) and toxic shock syndrome (TSS), and were characterized, among others, by fever, gastrointestinal complaints, and cardiac involvement. A number of these children required urgent intensive care treatment due to the development of multiorgan failure and circulatory shock, usually of myocardial origin. Some had evidence of macrophage activation syndrome (MAS). Laboratory abnormalities included markedly elevated acute phase reactants, increased ferritin and D-dimer, hypoalbuminemia as well as lymphopenia and relative thrombocytopenia. Patients with myocarditis had elevated levels of pro-B-type natriuretic peptide (proBNP) and troponin. Management was based on the administration of intravenous immunoglobulin (IV Ig) and glucocorticoids. In some instances, IL-1, IL-6 or tumor necrosis factor inhibitors were given. A temporal association with SARS-CoV-2 infection has been hypothesized because some children tested positive for the virus, either by reverse transcriptase-polymerase chain reaction (RT-PCR) or serology, or were exposed to potential contact with a household member affected with COVID-19.

The severity of this condition contrasted with the initial reports from China and Western countries, which had shown that relatively few children and adolescents were affected by COVID-19, and that most of those infected had experienced milder disease compared to adults (8–10). However, epidemiologic data indicated that the onset of MIS-C occurred 3–8 weeks after prior infection or known exposure, suggesting that SARS-CoV-2 acted as a trigger of a post-infectious inflammatory process (likely driven by some aspect of the adaptive immune system) (2, 11). Indeed, the vast majority (>80–90%) of children are positive for antibodies to SARS-CoV-2, whereas a smaller subset may be positive on RT-PCR for the virus. However, based on high cycle thresholds to detect the virus by RT-PCR in MIS-C patients, it is likely the virus is no longer infectious. In parallel with the rise in the number of articles describing the features of this condition in various parts of the world, an intense debate began regarding whether MIS-C and KD represent different illnesses with overlapping clinical features or are on the same disease spectrum. Although most experts favor the assumption that MIS-C is a novel entity with respect to KD (4, 11–17), some, including us, have argued that the two disorders may be a continuum, with some of the differences in phenotypic severity being due to the magnitude or kinetics of the immune response (18).

In the present viewpoint, we examine critically the evidence that supports the latter hypothesis and provide our interpretation of the relationship between the two conditions.

## Relationship Between KD and SARS-CoV-2

During COVID-19 pandemic a large number of children with classic or incomplete KD by the American Heart Association (AHA) criteria (19) were seen in affected countries. Many of these cases appeared to be related to SARS-CoV-2 infection, as they tested positive on either RT-PCR or serology. In Italy, most instances of KD occurred during the lockdown period imposed by public health authorities to contain the spread of the epidemic. Because in these months (March–May 2020) children stayed at home and neither attended school nor had social interactions with peers, they were likely not exposed to infectious agents other than the SARS-CoV-2. It is, thus, conceivable that at least a fraction of the cases of “genuine” KD seen during the pandemic were linked to SARS-CoV-2, as it has been ascertained for MIS-C. Furthermore, considering that KD has long been thought to be related to an infectious and/or environmental trigger, albeit still elusive (20), if one admits that SARS-CoV-2 did not play a causative role, a profound drop in the prevalence of KD should have been expected during the lockdown, owing to the lesser overall infectious morbidity in the pediatric population.

## Clinical Similarities Between KD and MIS-C

Although most children with MIS-C in the reported series did not fulfill the AHA criteria for KD, all had persistent fever and a variable proportion displayed one or more of the typical clinical manifestations of KD, namely rash, conjunctivitis, lips or oral changes, erythema/edema of the extremities, or cervical lymphadenopathy. Notably, the majority of children meeting the case definition for MIS-C seen at authors' hospital had conjunctival injection that spared the limbus, a sign characteristic of KD (19). These observations underscore the presence of many similarities in the clinical phenotype of the two conditions.

One of the arguments that are put forward to support the diversity between the two illnesses is that children with MIS-C have a high frequency of signs and symptoms that are unusual or occur rarely in KD, especially abdominal pain, vomiting, diarrhea, myocardial injury, and signs of meningeal irritation. However, all these features can be seen in classic KD. Vomiting, diarrhea and abdominal pain are part of the gastrointestinal symptoms of KD, together with hepatitis and hydrops of the gallbladder (16). Myocardial dysfunction occurs frequently in acute KD, and myocardial inflammation has been documented by scintigraphy in 50–70% of cases (19, 21). In addition, during the acute stage of KD, electrocardiography may show sinus and atrioventricular node functional abnormalities, with prolonged PR interval and non-specific ST and T-wave changes or low voltage (19). Note that pro-BNP and serum troponin, used in MIS-C to assess the severity of myocarditis, have been proposed as useful markers of cardiac involvement in KD (22, 23). Extreme irritability, exceeding that observed in other febrile illnesses, and aseptic meningitis are common neurologic findings of KD (19, 24). The development of coronary artery dilatation or aneurysms in some patients with MIS-C is difficult to reconcile with the hypothesis of the diversity of the two illnesses, as this complication has previously been attributed only to KD in pediatric patients.

As compared with KD, MIS-C is marked by more intense inflammation and by the frank tendency toward the development of shock and, to a lesser extent, MAS. However, TSS, which is seen in around 5% of children with KD (25), has many aspects in common with the shock syndrome of MIS-C. The development of MAS has been reported in 1–2% of cases of KD, but it is thought to be underrecognized (26). Thrombocytopenia is another feature of MIS-C that is not typical of KD, which is characterized by thrombocytosis. However, a drop in platelet count is frequently encountered in both KD-associated TSS and MAS. A further distinctive hematologic abnormality of MIS-C is lymphopenia, which is usually not observed in KD, and is a hallmark of severe COVID-19, although its pathophysiology is unclear (27). Hyponatremia, rhabdomyolysis, and image findings of corpus callosum inflammation can also occur in MIS-C.

There are several similarities in the therapeutic approach to MIS-C and KD. A high proportion (70–80%) of children with MIS-C have been treated initially with IVIG. Because this therapeutic intervention is part of the standard protocol for KD, its choice implies that many physicians who first saw these patients had the clinical impression of KD, although the choice of this intervention could be due to the aim to control a potentially infectious process or to possibly prevent acquired coronary artery changes. In case of non-response to IVIG, shock or organ-threatening disease, adjunctive therapy with glucocorticoids was usually given (28), which is analogous to the regimens proposed for IVIG-refractory KD (19, 29). The IL-1 inhibitor anakinra has been occasionally employed for treatment of MIS-C resistant to IVIG and glucocorticoids or impending MAS. This biologic medication is becoming increasingly more popular also for the management of KD after failure of IVIG and its efficacy in KD is being scrutinized in a phase IIa trial (30).

A further similarity between the two condition is the self-limited clinical course, which usually lasts 2–3 weeks. Recently, with improved recognition and treatment of MIS-C, the average hospital stay, even for patients presenting with shock, has decreased to nearly 5 days (31). Finally, the parallelism between MIS-C and KD has been underscored in the experience in Italy by their simultaneous disappearance after the regress of the pandemic, at the end of May 2020, and by their concurrent reemergence around 1 month after the resurgence of COVID-19, in the fall of 2020.

The main similarities and differences between KD and MIS-C are summarized in Table 1.

**Table 1:** Main similarities and differences between MIS-C and KD.

**Clinical manifestations common to both KD and MIS-C** Fever, skin rash, conjunctival injection, cervical adenopathy, lip and oral changes, swollen hands and feet, irritability
**Clinical manifestations frequent in MIS-C, but less common in KD** Abdominal pain, diarrhea, meningeal signs, myocarditis, MAS (1–2% in KD, but 20–30% in MIS-C), toxic shock syndrome (5–7% in KD, but 30–40% in MIS-C)
**Laboratory abnormalities seen in MIS-C, but not in KD** Lymphopenia, relative thrombocytopenia (with the exception of MAS and TSS, in which thrombocytopenia is frequent)
**Other similarities between MIS-C and KD** The vast majority of children with MIS-C were given initial treatment with IVIG
Glucocorticoids were effective in patients with IVIG resistance, myocarditis or major complications (TSS or MAS)
The IL-1 inhibitor anakinra is used in severe instances of both MIS-C and KD
Both MIS-C and KD pursue a self-limited course, with recovery within 2–3 weeks
Some children with MIS-C developed coronary aneurysms
Both MIS-C and KD occurred during the lockdown, in the spring of 2020, when children were likely not exposed to infectious agents other than SARS-CoV-2^*^
After the end of May 2020, after the abate of COVID-19 epidemic, MIS-C and KD disappeared simultaneously^*^
The second wave of COVID-19, in the fall of 2020, was accompanied by a resurgence of both MIS-C and KD^*^
**Main difference between MIS-C and KD** Children with MIS-C are older than those with KD(median age in MIS-C > 5 years vs. <5 years in KD)

*MIS-C, multisystem inflammatory syndrome in children; KD, Kawasaki disease; TSS, toxic shock syndrome; MAS, macrophage activation syndrome; IVIG, intravenous immunoglobulin*.

* *Observation made in Italy*.

## Why the Age of Children With MIS-C Is Higher Than That of Children With KD?

A feature that is pointed to as distinguishing MIS-C as a unique disease process vs. KD is that the median age of MIS-C cases was 9–10 years in the largest reported series (1–6, 32), whereas KD occurs predominantly in children 5 years of age or younger and has a peak incidence at around 10 months of age (33). There are, however, several reasons that may explain why younger children are more spared than older children and adolescents by COVID-19, in general, and, as a consequence, also by MIS-C.

In the first years of life, the immune system may be more “trained” to fight against viral infections owing to repeated vaccination procedures (34, 35). Notably, an amino acid sequence homology between glycoprotein components of SARS-CoV-2 and measles and rubella viruses has been identified. Using an antibody epitope prediction online tool, the homologous sequence appeared to have an epitope property and to be involved in antibody production. These findings have led to suggest that humoral immunity created through the measles, mumps and rubella (MMR) vaccine could provide a defense against COVID-19 (36). Younger children can also be protected against SARS-CoV-2 in virtue of a cross-reactive immunity induced after the encounter with other coronaviruses, which are a frequent cause of respiratory tract infections in preschool age.

Another potential explanation for the lower rates of SARS-CoV-2 infection in children is the lower expression of the cell surface enzyme angiotensin-converting enzyme 2 (ACE2), a receptor that has been proven to bind to SARS-CoV-2 spike protein and to promote internalization of the virus into human cells (37). In a recent study, children aged 4–9 years were found to have lower gene expression of ACE2 in nasal epithelial samples compared with older children, young adults, and adults (38). It should be recognized, however, that the vast majority of children infected with SARS-CoV-2 do not develop MIS-C irrespective of previous vaccination with MMR and that the lower expression of ACE2 may explain the lesser severity of COVID-19, but does not explain why some children develop MIS-C and some do not.

Notably, a recent study has suggested that ACE2 expression decreases with aging (39). It could be postulated that if an individual has higher ACE2 expression, even if the virus blocks ACE2 via binding, the amount of remaining ACE2 might still be significant. Given the antinflammatory role of ACE2, its higher expression may thus, provide protection against MIS-C.

The distinctive lesser susceptibility to develop MIS-C in the early ages might also be secondary to the immaturity of the immune system, which may be less able to mount a hyperinflammatory response or a cytokine storm syndrome (40).

Epidemiologic studies of MIS-C suggest that younger children are more likely to present with KD-like features, while older children are more likely to develop gastrointestinal symptoms (severe abdominal pain, vomiting, diarrhea), myocarditis and shock, and may be more likely to present with the features of MAS (17, 41). Notably, children presenting on the severe spectrum of classic KD with TSS or MAS are usually older and are boys, consistent with the demographic described in MIS-C (18). The coronary changes, when seen, seem also more likely to develop in the younger KD-like group.

## A Unique Disease Spectrum?

Of the 149 cases of inflammatory illness in children and adolescents registered in Italy between February and May 2020 (32), 96 met the AHA criteria for classic or incomplete KD, 10 met the same criteria plus the case definition for MIS-C, and another 43 only met only the case definition for MIS-C. The clinical diversities between patients with MIS-C and KD paralleled those observed in other series, and children with MIS-C tested positive for SARS-CoV-2 more frequently that those with KD. However, the occurrence of the two illnesses in the same population, during the same period of time, and when children were exposed to virtually no infectious agent other than the SARS-CoV-2 due to the lockdown confinement suggests, on epidemiological grounds, that they represent a disease continuum, with KD being at the more benign end of the spectrum and MIS-C at the most severe end.

## Proposed Common Pathophysiology of KD and MIS-C

The etiology of KD is unknown, but it is generally considered the consequence of an abnormal immune response, in genetically predisposed children, to infectious triggers entering through the upper respiratory tract. Multiple infectious agents have been suspected over the years, including respiratory RNA virus. In 2005, a group from New Haven (CT, USA) detected a novel human coronavirus, named New Haven coronavirus (HCoV-NH), in the respiratory secretions of 8 of 11 children with KD vs. 1 of 22 controls tested by RT-PCR. A serological test was not performed (42). Another study made in Japan evaluated the association between two different coronaviruses (HCoV-NL63 and HCoV-229E) and KD through serological tests. No difference in HCoV-NL63 antibody positivity was found between patients and controls on immunofluorescence assay, whereas antibody level for HCoV-229E was higher in patients with KD (43). Although the association between KD and the coronavirus family has not been confirmed in subsequent studies (19), these observations appear intriguing in the light of the possible relationship between KD and SARS-CoV-2.

As highlighted elsewhere (44), autoinflammatory diseases have taught us that many rheumatic conditions may represent syndromes rather than diseases. A variety of monogenic illness have, indeed, been found to mimic the clinical features of polyarteritis nodosa (45, 46), Behçet disease (47), rheumatoid factor positive polyarthritis (48), systemic juvenile idiopathic arthritis (49) and systemic lupus erythematosus (50). These observations indicate that many conditions that have been traditionally called “diseases” are actually “syndromes,” whose pathophysiology may be exemplified as a sort of funnel, that is, as a stereotyped way of reacting to multiple different etiologic factors in individuals possessing a particular genetic predisposition. The recognition of the etiology may be of paramount relevance for the treatment, as demonstrated by the dramatic effectiveness of anti-tumor necrosis factor agents in polyarteritis nodosa associated with ADA2 mutation (51).

**Figure 1:**
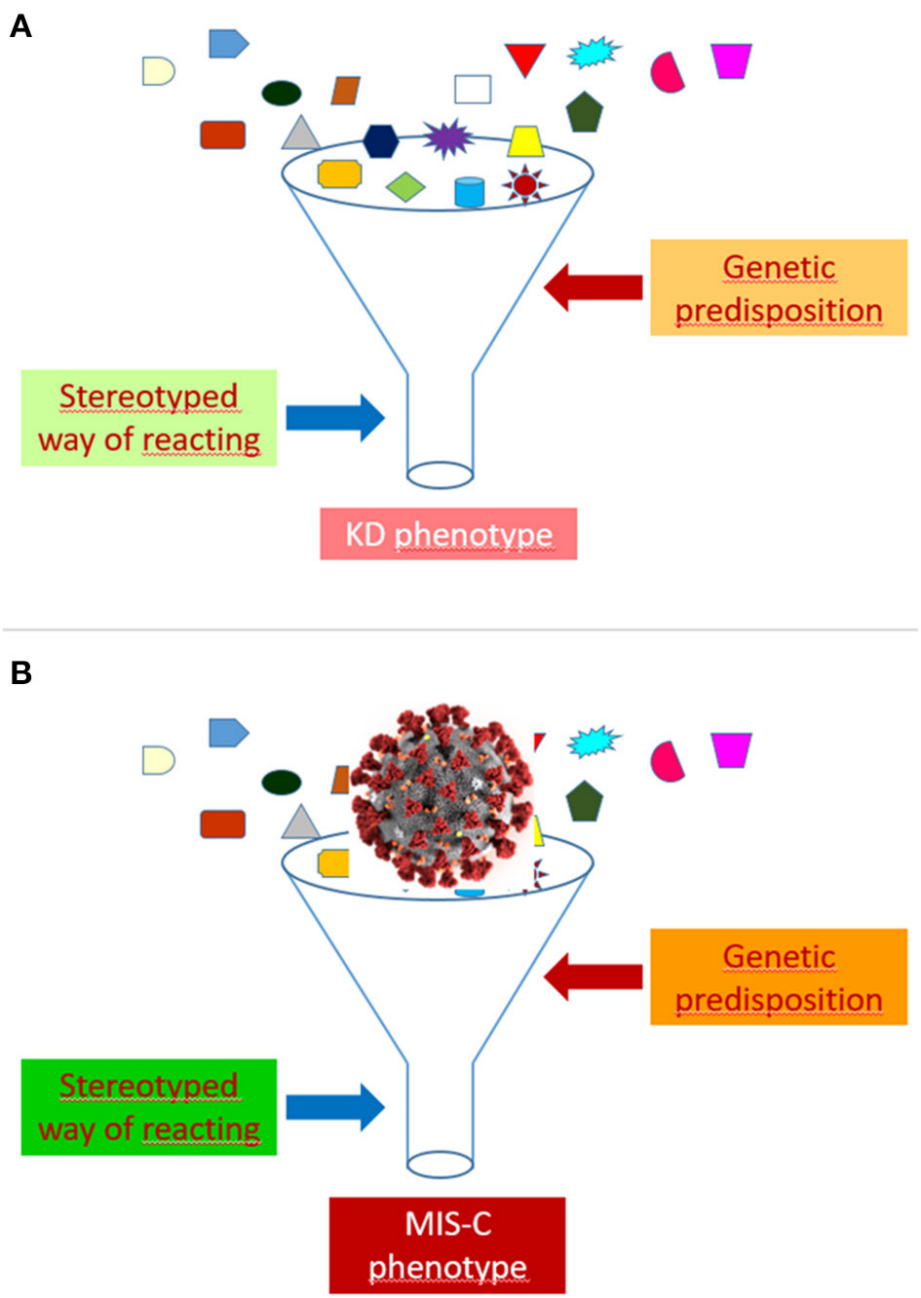
Funnel model of the pathophysiology of Kawasaki disease (KD) **(A)** and multisystem inflammatory syndrome in children (MIS-C) **(B)**.

In our view, the funnel model may be well-suited to illustrate the common pathophysiology of KD and MIS-C (Figure 1). In the case of MIS-C, an extremely aggressive and invasive virus like SARS-CoV-2, which has shown the capacity to cause a cytokine storm syndrome in adults with COVID-19 pneumonia, could induce, when presents with a massive viral load, a clinical phenotype much more inflammatory and acute than that of KD, and marked, in addition to the typical manifestations of KD, by a higher frequency of less common or atypical disease manifestations and of serious complications, such as myocarditis, TSS and MAS.

The development of a KD or a MIS-C phenotype after the contact with SARS-CoV-2 might depend on several factors, including, but not limited to, viral load, virulence of viral strains, child age, intensity or kinetics of the immune response, ethnic or socio-economic factors, comorbidities (especially obesity), and genetic background. Differences between patients in the viral burden could account for the more frequent positivity of SARS-CoV-2 tests in more severe cases.

Henoch-Schönlein purpura (HSP), the most common vasculitis in children, is also a syndrome. It is often preceded by a respiratory tract infection and multiple case studies have suggested a correlation with virtually all respiratory pathogens (52). Although usually benign, it can occasionally cause severe involvement of the kidney, gastrointestinal tract and central nervous system. Like KD, the pathophysiology of HSP appears consistent with an abnormal and stereotyped immune reaction to an infectious agent in genetically predisposed individuals. Although rather different, HSP has a self-remitting course, like KD.

Although the genetic determinants of KD are still elusive, that susceptibility is shaped by genetic influences is suggested by the preferential involvement of males, the predilection for particular racial/ethnic groups, with an incidence in Japan, Chorea and Taiwan 10- to 20-fold higher than in the United States, and the observation of an increased risk in family members and twins (19). The hypothesis of a common predisposing background between KD and MIS-C is corroborated by the report that 2 of 28 patients with MIS-C had KD in the past (53).

The role of genetic determinants in the induction of MIS-C vs. KD is highlighted by the marked epidemiologic differences among ethnic groups. In the United States, MIS-C had a greater impact on children of Afro-American and Hispanic ethnicity (5, 6) as in France and UK, were children of Afro-Caribbean descent were particularly hit (1, 3, 54). Conversely, MIS-C was apparently not observed in Japan and Chorea, countries characterized by a markedly elevated prevalence of KD. Despite a very high impact of COVID-19, MIS-C was not reported in China. These issues may lead to speculate that subjects belonging to ethnic groups less affected by classic KD may be distinctly susceptible to develop a more aggressive phenotype of KD, including MIS-C.

That a shared genetic background may underlie a continuum of inflammatory disorders of varied severity has been suggested by the detection of genetic similarities among recurrent aphthous stomatitis, periodic fever, aphthous stomatitis, pharyngitis, and cervical adenitis (PFAPA) syndrome, and Behçet disease. The genotypic overlap places these disorders on a common spectrum, with recurrent aphthous stomatitis on the mild end, Behçet disease on the severe end, and PFAPA intermediate (55).

Recent studies suggest that a defective antiviral response may be contributory in some patients with COVID-19. Inborn errors of type I interferon immunity and auto antibodies against type I interferons have been discovered in the most severe cases of COVID-19 (56, 57). It is, thus, conceivable that host immune dysregulation, as well as a molecular mimicry between SARS-CoV-2 and self-antigens, may be involved in the induction of the severe inflammatory manifestations of MIS-C.

The inflammatory response in MIS-C was found to share several features with KD, but also to differ from this condition in the proportion of particular subsets of T-lymphocytes, the characteristics of IL-17A-mediated immunopathology, the concentration of biomarkers of arterial inflammation and damage, and the profile of autoantibodies to proteins involved in immune response or to structural components of heart and blood vessels (58). However, the meaning of this study is affected by the choice of contrasting children with MIS-C with a historical cohort of KD seen before COVID-19 pandemic. In our view, the comparison of patients with the features of MIS-C and KD seen during the course of the pandemic could provide better insights into the relationship between the two conditions, particularly in the light of their common relationship with SARS-CoV-2.

## Conclusions and Future Directions

Based on the above considerations, we favor the hypothesis that MIS-C is on the KD spectrum, instead of representing a new childhood inflammatory disorder separate from KD. The occurrence of a KD-like condition in association with SARS Cov-2 infection underscores the notion that KD is not a disease, but rather a syndrome, whose main features and phenotypic severity depend on the magnitude and type of the immune response as well as on the characteristics of the host and of the triggering infectious agent (44). Notably, the interpretation of KD as a syndrome is in keeping with the first description by Tomisaku Kawasaki, who called it “acute febrile mucocutaneous lymph node syndrome” (59, 60). It should be recognized, however, that there are still limited data on MIS-C, particularly regarding well-established diagnostic criteria, pathophysiology, and outcome information. Thus, further studies of the genetic and immunopathologic background are required to establish more precisely the relationship between MIS-C and KD. More in general, the spectrum of pathology that has emerged during the pandemic offers a unique opportunity for investigations aimed to elucidate the pathophysiology not only of KD, but also of other inflammatory disorders whose causative factors and mechanisms are still unknown.

## Data Availability Statement

The original contributions presented in the study are included in the article/supplementary materials, further inquiries can be directed to the corresponding author/s.

## Author Contributions

All authors listed have made a substantial, direct and intellectual contribution to the work, and approved it for publication.

## Conflict of Interest

The authors declare that the research was conducted in the absence of any commercial or financial relationships that could be construed as a potential conflict of interest.
